# Induction of Protective Immunity by a Single Low Dose of a Master Cell Bank cGMP-rBCG-P Vaccine Against the Human Metapneumovirus in Mice

**DOI:** 10.3389/fcimb.2021.662714

**Published:** 2021-06-29

**Authors:** Jorge A. Soto, Nicolás M. S. Gálvez, Gaspar A. Pacheco, Gisela Canedo-Marroquín, Susan M. Bueno, Alexis M. Kalergis

**Affiliations:** ^1^ Millennium Institute on Immunology and Immunotherapy, Departamento de Genética Molecular y Microbiología, Facultad de Ciencias Biológicas, Pontificia Universidad Católica de Chile, Santiago, Chile; ^2^ Departamento de Endocrinología, Facultad de Medicina, Pontificia Universidad Católica de Chile, Santiago, Chile

**Keywords:** hMPV, human metapneumovirus, innate immunity, adaptive immunity, vaccine, recombinant BCG, master cell bank cGMP

## Abstract

Human metapneumovirus (hMPV) is an emergent virus, which mainly infects the upper and lower respiratory tract epithelium. This pathogen is responsible for a significant portion of hospitalizations due to bronchitis and pneumonia in infants and the elderly worldwide. hMPV infection induces a pro-inflammatory immune response upon infection of the host, which is not adequate for the clearance of this pathogen. The lack of knowledge regarding the different molecular mechanisms of infection of this virus has delayed the licensing of effective treatments or vaccines. As part of this work, we evaluated whether a single and low dose of a recombinant *Mycobacterium bovis* Bacillus Calmette-Guérin (BCG) expressing the phosphoprotein of hMPV (rBCG-P) can induce a protective immune response in mice. Immunization with the rBCG-P significantly decreased neutrophil counts and viral loads in the lungs of infected mice at different time points. This immune response was also associated with a modulated infiltration of innate cells into the lungs, such as interstitial macrophages (IM) and alveolar macrophages (AM), activated CD4^+^ and CD8^+^ T cells, and changes in the population of differentiated subsets of B cells, such as marginal zone B cells and plasma cells. The humoral immune response induced by the rBCG-P led to an early and robust IgA response and a late and constant IgG response. Finally, we determined that the transfer of cells or sera from immunized and infected mice to naïve mice promoted an efficient viral clearance. Therefore, a single and low dose of rBCG-P can protect mice from the disease caused by hMPV, and this vaccine could be a promising candidate for future clinical trials.

## Introduction

Human metapneumovirus (hMPV) is considered the second most prevalent virus associated with acute lower respiratory tract infections (ALRTI) worldwide in newborns, children under five years of age, and the elderly (Boivin et al., 2003). An increase in the infection rates of this emerging virus has been recently reported as compared to other common respiratory viruses, such as adenovirus and parainfluenza A (Edwards et al., 2013). The observed increase could be due to an improvement in the techniques used to diagnose and detect hMPV (Edwards et al., 2013). However, the lack of knowledge underlying the evasion mechanism used by hMPV to avoid the immune system has delayed the development of therapies to control the infection caused by this virus.

hMPV belongs to the *Pneumoviridae* family and the *Metapneumovirus* genus. The genome of this virus is a negative-sensed and single-stranded RNA (ssRNA) of about 13 Kb, which encodes for eight genes including nine structural proteins in the following order: 3**’**-Nucleoprotein(N)-Phosphoprotein(P)-Matrix protein(M)-Fusion protein (F)-M2.1-M2.2-Small Hydrophobic protein (SH)-Glycoprotein (G)-Large RNA-dependent RNA Polymerase (L)- 5**’** (van den Hoogen et al., 2002). The F, G, and SH proteins are found on the surface of the viral particle. The G and F proteins are involved in the attachment and entry of the virus into the host cell (Terrier et al., 2008). The G protein is a type II transmembrane glycoprotein that is commonly associated with the attachment of the viral particle into the host cell (Thammawat et al., 2008). Although the G protein is not essential during viral replication *in vitro*, viral replication *in vivo* is attenuated in G mutant strains (Biacchesi et al., 2004; Biacchesi et al., 2005). The fusion protein is a homo-trimeric class I fusion glycoprotein typically found in pneumoviruses, such as hRSV. This protein undergoes a proteolytical cleavage from a precursor form (F_0_) to reach a final conformation, interacting with the host cell (Smith et al., 2009). The F protein alone could be enough to promote the fusion and infection of the virus into the host cell in the absence of the G protein. This capacity could be associated with a specific arginine-glycine-aspartate (RGD) motif localized in some integrins (Cox et al., 2012). The SH protein is a type II transmembrane glycoprotein of 179 amino acids, characterized as different forms depending on the glycosylation pattern (Biacchesi et al., 2005), and it is a suggested viroporin that affects the permeability of the cell membrane for compounds, such as hygromycin B (Masante et al., 2014).

The M protein is involved in the encapsidation and budding of new viral particles, as it composes the matrix of the virus (van den Hoogen et al., 2002; Leyrat et al., 2014). This protein has been characterized to promote dendritic cell (DC) activation *in vitro* (Soto et al., 2020). Also, the dimeric form of the M protein showed a high-affinity Ca^2+^ binding site (Leyrat et al., 2014). The N protein coats and protects the viral RNA while synthesized and regulates viral replication and transcription (van den Hoogen et al., 2002; Derdowski et al., 2008). A functional N protein conformation depends on the binding of several monomeric N-proteins, allowing the correct interaction of the RNA with this structure (Renner et al., 2016). The N, P and L proteins are part of a protein complex that replicates and transcribes the genetic material (Easton et al., 2004). Lastly, the M2.1 and M2.2 proteins work as cofactors to activate the above-mentioned complex (Buchholz et al., 2005).

The Phosphoprotein (P) is an essential cofactor of the viral RNA-dependent RNA polymerase (Easton et al., 2004). Furthermore, the P protein interacts directly with the N protein, allowing an efficient regulation of the N protein to discriminate the correct RNA strands to bind (Renner et al., 2016). The interaction between the P and the N proteins promotes the formation of inclusion bodies onto the cells (Derdowski et al., 2008) and stimulates a reorganization of the actin present on a direct cell-to-cell viral spread (El Najjar et al., 2016). The P protein has also been described as an immunodominant target during viral infection, promoting the expansion of CD4^+^ and CD8^+^ T cells that produce IFN-γ (Tzannou et al., 2017).

The natural immune response elicited by hMPV infection is complex and insufficient to achieve viral clearance (González et al., 2017; Gálvez et al., 2021). This immune response is frequently linked to a pro-inflammatory environment in the host, followed by a strong Th2-like immune polarization of the responding CD4^+^ T cells (González et al., 2017). An adequate antiviral response would require a Th1 immune polarization of the T cell response (González et al., 2017). The inadequate immune response induced upon a natural infection with hMPV leads to an increase in the secretion of several cytokines, such as Interleukin (IL)-4, IL-5, and IL-8 (Ito et al., 2004; Romagnani, 2006; Fietta and Delsante, 2009), along with an increase in the infiltration of polymorphonuclear (PMN) cells into the lungs and a defective T cell immune response (Ito et al., 2004; Romagnani, 2006; Fietta and Delsante, 2009; Céspedes et al., 2013). This inadequate T cell response is associated with an inappropriate activation of these lymphocytes by hMPV-infected antigen-presenting cells (APC), such as DCs (Céspedes et al., 2013). Moreover, the infection caused by hMPV has been associated with increased mucus production and the subsequent obstruction of the airways (Boivin et al., 2003; Hamelin et al., 2006; Céspedes et al., 2013).

To counteract the adverse effects caused by hMPV, vaccines with different action mechanisms have been developed (Liu et al., 2013; Lévy et al., 2013; Cox et al., 2014; Karron et al., 2017; Diaz-Dinamarca et al., 2018). Among these vaccines are live-attenuated virus (Liu et al., 2013; Karron et al., 2017), recombinant proteins -such as the N-hMPV protein formulated with the AbISCO-100 adjuvant- (Diaz-Dinamarca et al., 2018), Virus-Like Particles (VLPs) (Lévy et al., 2013; Cox et al., 2014), and mRNA vaccines that are currently being tested in clinical trials (clinicaltrials.gov. NCT04144348 and NCT03392389). However, none of these strategies are commercially available for human use up to date. Accordingly, neither vaccines nor prophylactic methods (such as monoclonal antibodies) specific for hMPV are currently available.

We have previously generated and evaluated a recombinant *Mycobacterium bovis* Bacillus Calmette-Guérin (BCG) that expresses the phosphoprotein of hMPV (rBCG-P), which is found among the current vaccines candidate against this virus (Palavecino et al., 2014; Soto et al., 2018; Andrade et al., 2020). The cellular and the humoral response elicited upon immunization with this vaccine in mice has been previously described (Palavecino et al., 2014; Soto et al., 2018). Immunization with the rBCG-P vaccine induces an enhanced antiviral immune response, associated with the proliferation of IFN-γ-expressing CD4^+^ and CD8^+^ T cells (Palavecino et al., 2014) and the promotion of a protective immunity mediated by antibodies specific against the virus and several of its proteins through a process commonly known as linked recognition (Soto et al., 2018). The immunization scheme performed in previous reports considered an initial vaccination and a posterior boost, both with an immunization dose of 1x10^8^ CFU (Palavecino et al., 2014; Soto et al., 2018). Here, we evaluated the immune response and the protection elicited by an rBCG-P strain generated under the initial phases of a current Good Manufacturing Practices (cGMP-master cell bank) process during infection with hMPV. Using only a single and low dose (1x10^5^ CFU, which is the current dose of BCG administered to humans), we found that the immune response induced by the rBCG-P vaccine decreased the disease parameters and protected mice from severe disease caused by this virus. This protection lasted at least up to 28 days post-infection in mice, exhibiting a well-balanced response between cellular and humoral immunity. Therefore, the rBCG-P vaccine is a viable option to advance further into clinical trials and eventually be used as an immunization strategy against hMPV.

## Materials and Methods

### hMPV Propagation and Titration

LLC-MK2 cells (American Type Culture Collection, CCL-7™) were used to propagate hMPV serogroup A, strain CZ0107 (clinical isolate obtained from the *Laboratorio de Infectología y Virología* of the *Hospital Clínico, Pontificia Universidad Católica de Chile*) (Reina et al., 2007). Cells were cultured in T75 flasks with a growth medium (Opti-MEM supplemented with 5% FBS) until 80-90% confluence. Then, cells were infected with hMPV at an MOI equal to 0,1 in 5 mL of infection medium (Opti-MEM supplemented with 100 µg/ml CaCl_2_ and Trypsin 5 µg/mL) and incubated at 37°C for 2 hours. The supernatant was then replaced with a fresh infection medium and incubated for 72 h or until an evident cytopathic effect was observed. After this time, cells were scraped, and the content of the flask was pooled and centrifuged first at 300 x g for 10 min and then at 500 x g for 10 min to remove cell debris. Between centrifugations, cells were disaggregated by pipetting. Supernatants of non-infected LLC-MK2 cells were collected as previously described and used as non-infectious control (Mock) in the following procedures. Viral titers of supernatants were determined by immunocytochemistry in 96-well plates with LLC-MK2 cells, as previously described (Reina et al., 2007; Tollefson et al., 2010; Céspedes et al., 2013; Espinoza et al., 2013).

### Doses of BCG-WT and rBCG-P for Immunization

Vaccine doses of BCG-WT (Danish 1331 strain) were generated as previously described (Bueno et al., 2008; Palavecino et al., 2014). Briefly, BCG-WT was prepared by growing the microorganism on 7H9 liquid medium (Sigma-Aldrich, M0178-500G), supplemented with 10% OADC (Sigma-Aldrich, M0678-1VL) until reaching an OD600 of 0.8. Then, the mycobacterial culture was washed three times with PBS plus 0.05% Tween 80, resuspended with PBS plus glycerol 50% at a final concentration of 4x10^5^ CFU per vial and frozen at -80°C until their posterior use. The master cell bank batch of the rBCG-P vaccine was manufactured under good manufacturing practices (cGMP) in IDT Biologika (USA). The vaccine growth process was performed in strictly controlled conditions, without supplementation with OADC and stored at -80 °C after their generation. The expression of the P protein by this strain was routinely confirmed by dot blot and western blot assays and the presence of the gene was confirmed by PCR. For immunization, vials were centrifuged at 14,000 x g for 15 minutes and resuspended in saline solution to a final concentration of 1x10^5^ CFU per 100 μL before injection. Plating of resuspended vials was routinely performed to corroborate the CFU value administered.

### Mouse Immunization and Viral Infection

Six- to eight-week-old BALB/cJ mice received a subcutaneous injection in the right dorsal flank consisting of 1 x 10^5^ CFU of either BCG WT or rBCG-P in a final volume of 100 μL per dose (n=4 per group). Twenty-one days post-immunization, mice were intraperitoneally anesthetized with a mixture of ketamine and xylazine (80 mg/kg and 4 mg/kg, respectively), and this anesthesia stock was diluted 3 fold in physiological serum before the challenged with 1 × 10^6^ PFU of hMPV A (strain CZ0107) by intranasal instillation, in a final volume of 100 mL per mouse. Blood samples were obtained from these animals before immunization and challenge. Blood samples were also obtained 7 days post-infection (dpi) and 28 dpi. Lung samples and bronchoalveolar lavages (BAL) were obtained at 7 dpi and 28 dpi to evaluate cellular infiltration upon reaching the protocol endpoint and performing euthanasia to the corresponding mice. Additionally, T and B cell populations were also measured from lung and BAL samples.

The following clinical parameters were evaluated considering these scores: 1) weight loss (0 = no weight loss; 1= <10%; 2 = 10% to 20%; 3 = 20% to 25%. Animals with a weight loss higher than 25% are euthanatized). 2) Animal posture and fur (0= normal upright and no ruffle; 1= partially curved and ruffled; 2= curved and significantly ruffled; 3= prostrate or immobile and significantly ruffled (euthanasia). 3) Response towards stimuli (0= normal, aware of the environment; 1= slightly reduced movement and grooming; 2= wobbly scrolling; 3= idle at the bottom of the cage). 4) Adverse reaction at the immunization site (0= no changes in the area; 1=mild dermatitis (erythema/pruritus); 2= dermatitis with alopecia and pain on contact; 3= ulcerative dermatitis).

All mouse experiments were conducted in agreement with ethical standards and according to the local animal protection law number 20.800. All experimental protocols were designed according to the Sanitary Code of Terrestrial Animals of the World Organization for Animal Health (OIE, 24^a^ Edition, 2015) and were reviewed and approved by the Scientific Ethical Committee for Animal and Environment Care of the Pontificia Universidad Católica de Chile (Protocol number 160819006).

### Quantification of IgG isotypes and IgA Measurement

To quantify IgG or IgA antibodies in the samples obtained, 96-well ELISA plates were coated overnight at 4°C with 1 x 10^6^ PFU on 50 μL/well of hMPV (previously UV-inactivated for 45 minutes and heat-inactivated for 15 minutes at 80°C to expose as many antigens as possible), or 100 ng on 50 µL/well of purified P-hMPV protein. Plates were blocked with 200 μL of PBS plus 5% BSA (Winkler BM-0150) for one h at RT. Then, plates were washed three times with 200 μL of PBS plus 0.05% Tween 20 and incubated for one h at RT with 100 μL of the different serum samples (dilution 1/100) to measure IgG, or BAL samples (undiluted) to measure IgA. These measurements were performed with the 7 dpi and 28 dpi samples. Then, plates were washed three times and incubated with 50 μL of 1/2,000 dilution of HRP-Goat anti-mouse IgG (H+L) (Life Technologies, N. Meridian rd., Rockford, IL 61101, USA) or HRP-Goat anti-mouse IgA (Life Technologies, N. Meridian rd., Rockford, IL 61101, USA) for one h at RT. Finally, plates were washed three times with 200 μL of PBS plus 0.05% Tween 20, and one time with PBS. Plates were revealed with 50 μL of 1 mg/ml 3,3’,5,5’-tetramethylbenzidine (TMB, BD Biosciences) at RT protected from the light for 15 minutes, and the reaction was stopped with the addition of 50 μL of 2N H_2_SO_4_ solution. Plates were analyzed in an ELISA reader at 450 nm (Multiskan Ex, Thermo Labsystems).

### Splenocyte Culture, T Cell Transfer, and Total IgG Transfer to Naïve Mice

Spleens were recovered from euthanized mice at 7 dpi from the following groups: non-immunized and hMPV-infected (hMPV), BCG-WT immunized and hMPV-infected (BCG-WT + hMPV), rBCG-P immunized, and hMPV-infected (rBCG-P + hMPV). Spleens were disaggregated and incubated for 5 min at RT with 500 μL of Ammonium-Chloride-Potassium (ACK) lysis buffer to lysate red cells and centrifuged at 300 x g for 5 min at 4°C. Supernatants were discarded, and cells were washed with 1 mL of sterile PBS. Then, cells were resuspended in 5 mL of RPMI 1640 media supplemented with 10% FBS (Andes Import). A 10 µL aliquot of each spleen was mixed with trypan blue stain in a 1:1 dilution (Gibco, Thermo Fischer) to determine the number of cells obtained. A live-dead quantification was performed to ensure optimal conditions of the culture (data not shown). 2 x 10^6^ cells were cultivated in 24-well plates and incubated at 37°C with 5% CO_2_ for 48 hours. For stimulation, 1 μg of purified hMPV-P protein was used in each well. A mixture of α-CD3 and α-CD28 was used as a positive control for T cell activation because this stimulus promotes a specific activation and proliferation of T cells by directly engaging their TCR/CD3 complex (Thaker et al., 2015). After 48 hours of culture, cells were pooled and then purified by MACS column (Miltenyi Biotec). Total T cells were purified by positive selection using columns and buffers provided by the manufacturers as previously described (Cat. #130-095-130, Miltenyi Biotec). Finally, T cells were kept at 4°C until they were used in passive transfer assays, administered two hours later by the lateral tail vein. Confirmation of T cell purification was routinely performed by flow cytometry (data not shown).

For total IgG transfer, sera samples used were from the time of euthanasia of the following groups: hMPV, BCG-WT + hMPV, rBCG-P + hMPV (n=4 for each group).

Six- to eight-week-old naïve BALB/cJ mice were intravenously transferred with 100 μg of total IgG. The IgG concentration was determined by ELISA, considering a standard curve for interpolation. Likewise, whole T cells obtained from splenocyte cultures were also used to transfer different groups of naïve mice (1x10^6^ T cell per mice). Mice weight mainly was uniform among the animals.

One day after sera or T cell transfer, animals were intraperitoneally anesthetized with a mixture of ketamine and xylazine (80 mg/kg and 4 mg/kg, respectively) and challenged by intranasal instillation with 1 x 10^6^ PFU of hMPV. As negative and infection controls, one group was treated with non-infectious supernatant (mock), and one group was not transferred but was still infected with hMPV, respectively. Parameters such as weight loss and clinical score were registered daily. At 7 dpi, animals were terminally anesthetized by i.p. injection with a mixture of ketamine/xylazine (80mg/kg and 4mg/kg, respectively). BAL and lung samples were collected from these mice to evaluate various parameters of infection and disease.

### Evaluation of hMPV-Associated Disease Parameters

To characterize the infiltration into the lungs of polymorphonuclear (PMN), T, and B cells, BAL and lung samples were collected as previously described (Bueno et al., 2008) and as indicated above. Then, samples were stained with the following antibodies to evaluate myeloid populations: α-CD45-BV510 (clone 30-FL1, BD Horizon); α-CD11b PerCP-Cy 5.5 (clone M1/70, BD Pharmingen), α-CD11c APC (clone HL3, BD Pharmingen), and α-Ly6G FITC (clone 1A8, BD Pharmingen). The following antibodies were used to evaluate lymphoid populations: α-CD45-BV510 (clone 30-FL1, BD Horizon); α-CD3/TCRβ-FITC (clone H57-597, BD Pharmigen); α-CD4-APC/H7 (clone RPA-T4, BD Pharmigen); α-CD8-PE/Cy7 (clone RPA-T8, BD Pharmigen); α-CD69- APC (clone H1.2F3, BD Pharmigen); α-CD9-BV786 (clone KHC8, BD OptiBuild); α-CD19-BUV737 (clone SJ25C1, BD Horizon); α-CD21/35-APC (clone 7G6, BD Pharmigen); α-IgM-PEcf594 (clone R6-60.2, BD Horizon); α-B220-APC/H7 (clone RA3-6B2, BD Pharmigen); α-CD11b-PerCP-Cy 5.5 (clone M1/70, BD Pharmingen); α-CD11c-APC (clone HL3, BD Pharmingen); and α-IA/IE-V500 (clone M5/114.15.2, BD Pharmigen). Data were acquired in an LSRFortessa X2-0 cytometer (BD Biosciences) and analyzed using FlowJo v10.0.7 software (BD Biosciences). All analyses were performed on CD45^+^ cells. Viral loads were detected in the lungs by qRT-PCR as previously described (Bueno et al., 2008; Palavecino et al., 2014). Lung samples for histopathological analysis were stored in 4% Paraformaldehyde (PFA), maintained at 4°C, embedded in paraffin, cut, and stained with Hematoxylin & Eosin (H&E) as previously described (Bueno et al., 2008). The histopathological score quantification was evaluated as previously described (González et al., 2021).

### Gene Expression Evaluation by RT-qPCR

Lung samples were homogenized in TRIzol solution (ThermoFisher), and RNA extraction was performed according to the instructions given by the manufacturer. Briefly, lungs were disaggregated in 1 mL of TRIzol, and 200 µL of chloroform were added. Tubes were mixed by inversion and centrifuged at 12,500 x g for 15 minutes at 4°C. The upper aqueous layer was collected, and 250 µL of cold isopropyl alcohol were integrated into the collected phase. After 15 minutes of incubation in ice, tubes were centrifuged at 10,000 x g for 10 minutes at 4°C. Supernatants were discarded, and RNA pellets were washed with 1 mL of cold, nuclease-free 75% aqueous ethanol solution. Tubes were centrifuged at 7,500 x g for 5 minutes at 4°C. Supernatants were discarded, and pellets were dried before dissolving them in 20 µL of nuclease-free, DEPC-treated water. Samples were frozen at -80°C until further analysis. RNA concentration was determined by UV spectrophotometry in a NanoDrop 2000 Spectrophotometer (ThermoFisher). cDNA was synthesized using an iScript cDNA Synthesis kit (ThermoFisher) using 1 µg of total RNA, according to the instructions given by the manufacturer. cDNA was stored at -20°C until further analysis. The qPCR reaction was performed using SYBR Green reagents (BioRad), a final concentration of 200 nM for each primer, and 1 µL of sample cDNA as a template. Real-time PCR was performed in a StepOnePlus Real-Time PCR System (ThermoFisher). Samples were heated at 95°C for 5 minutes, followed by 40 cycles consisting of 5 seconds at 95°C and 30 seconds at 60°C. The primers used for each target gene are described in **Table 1**. The values obtained were normalized using the housekeeping β-actin gene.

**Table 1:** List of primers used to evaluate expression of target genes.

Target	Forward primer	Reverse primer	PrimerBank ID
**IL-1β**	5’ GCA ACT GTT CCT GAA CTC AAC T 3’	5’ ATC TTT TGG GGT CCG TCA ACT 3’	6680415a1
**IL-4**	5’ GGT CTC AAC CCC CAG CTA GT 3’	5’ GCC GAT GAT CTC TCT CAA GTG AT 3’	10946584a1
**IL-6**	5’ TAG TCC TTC CTA CCC CAA TTT CC 3’	5’ TTG GTC CTT AGC CAC TCC TTC 3’	13624311a1
**IL-10**	5’ GCT CTT ACT GAC TGG CAT GAG 3’	5’ CGC AGC TCT AGG AGC ATG TG 3’	6754318a1
**IL-12p40**	5’ ACA GGT GAG GTT CAC TGT TTC T 3’	5’ TGG TTT GCC ATC GTT TTG CTG 3’	6680397a1
**IL-17A**	5’ TTC CTC CAC TGA TCC TTG TTC T 3’	5’ AGC AGC TTC CTT TGT ATC ATC AC 3’	6680433a1
**IFN-α**	5’ TCC TGA ACC TCT TCA CAT CAA A 3’	5’ ACA GGC TTG CAG GTC ATT GAG 3’	**-**
**IFN-β**	5’ AGC TCC AAG AAA GGA CGA ACA 3’	5’ GCC CTG TAG GTG AGG TTG AT 3’	6754303c1
**IFN-γ**	5’ ACA GCA AGG CGA AAA AGG ATG 3’	5’ TGG TGG ACC ACT CGG ATG A 3’	145966741c2
**TNF-α**	5’ CAG GCG GTG CCT ATG TCT C 3’	5’ CGA TCA CCC CGA AGT TCA GTA G 3’	133892368c1
**TGF-β1**	5’ CTC CCG TGG CTT CTA GTG C 3’	5’ GCC TTA GTT TGG ACA GGA TCT G 3’	6755775a1
**CCL2**	5’ GCC GAT GAT CTC TCT CAA GTG AT 3’	5’ GCA TTA GCT TCA GAT TTA CGG GT 3’	6755430a1
**CXCL13**	5’ GGC CAC GGT ATT CTG GAA GC 3’	5’ GGG CGT AAC TTG AAT CCG ATC TA 3’	9256521a1
**CX3CL1**	5’ ACG AAA TGC GAA ATC ATG TGC 3’	5’ CTG TGT CGT CTC CAG GAC AA 3’	31982017a1
**β-actin**	5’ GGC TGT ATT CCC CTC CAT CG 3’	5’ CCA GTT GGT AAC AAT GCC ATG T 3’	6671509a1
**N-hMPV**	5’ ACA GCA GAT TCT AAG AAA CTC AGG 3’	5’ TCT TTG TCT ATC TCT TCC ACC C 3’	–

C_T_ values were used for calculation of relative gene expression by the double delta C_T_ method (2^-ΔΔCt^), using β-actin as a calibrator gene.

### Quantification of Cytokine Concentration by ELISA

The concentration of several cytokines was determined via ELISA in serum and lung supernatant samples obtained from mice at 7 dpi and 28 dpi after the homogenization of the lung. The following cytokine ELISA kits were used to determine cytokine concentrations: IFN-γ (BD Biosciences, Cat. # 555138, Lot 0148738), IL-1β (BD Biosciences, Cat. # 559603, Lot 4056838), IL-2 (BD Biosciences, Cat. # 555148, Lot 0149962), IL-4 (BD Biosciences, Cat. # 555232, Lot 0070759), IL-6 (BD Biosciences, Cat. # 555240, Lot 7333548EU), IL-10 (BD Biosciences, Cat. # 555252, Lot 9164829), TNF (BD Biosciences, Cat. # 555268, Lot 7040549). All procedures for each ELISA determination were performed according to the instructions given by the manufacturer. Overall, ELISA plates were incubated with 50 µL of undiluted samples for 1 hour. Plates were washed in an ELx50 96-well plate washer (BioTek), and absorbance was measured in an 800 TS Microplate Reader (BioTek). Absorbance measurements at a 562 nm wavelength was subtracted to the absorbance measurement at a 450 nm wavelength when analyzing optical density data. A standard curve was included for each cytokine kit, and the concentration of each cytokine in lung and serum was interpolated from the lineal fit for each curve.

### Statistical Analyses

All statistical analyses were performed using GraphPad Prism version 9.1.0 Software. Statistical differences were assessed through a one-way or two-way ANOVA followed by a *post hoc* Tukey test, as indicated in the legend of each figure. Differences between means were considered to be statistically significant when the calculated P-value was <0.05. Given the small sample sizes, data are presented as mean ± standard error of the mean (SEM) to represent the accuracy of the estimation of our sample means to the actual population mean. The significance for each statistic test is shown in the legend of each figure.

## Results

### A Single and Low Dose of a Master Cell Bank cGMP rBCG-P Vaccine Can Decrease the Disease Parameters Caused by hMPV Infection

**Figure 1:**
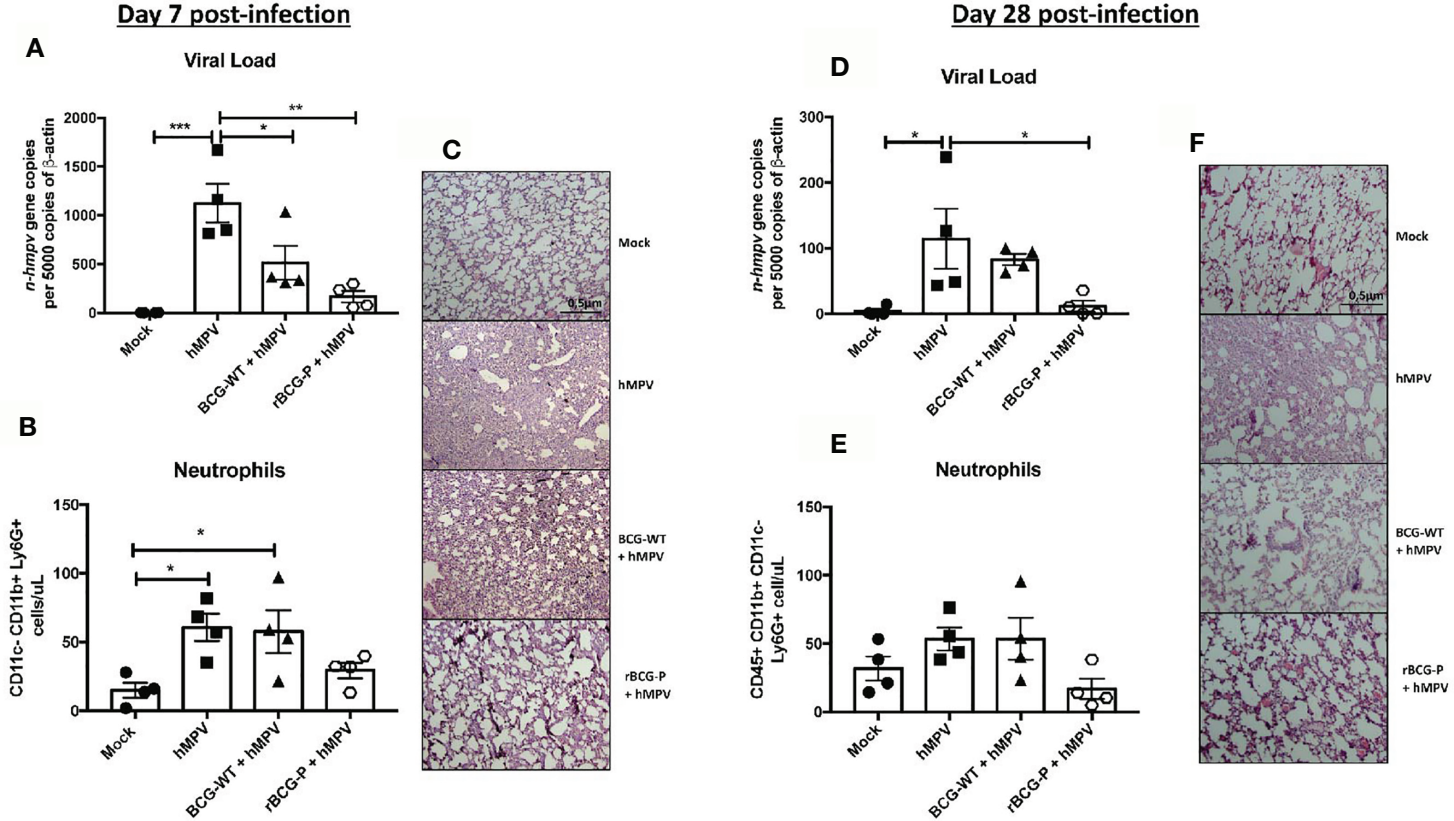
Evaluation of disease parameters on hMPV-infected animals at 7- and 28-days post-infection. Disease parameters were measured in the mice infected with hMPV to evaluate the protective capacity of the rBCG-P vaccine. All the parameters were measured at day 7 **(A–C)** and 28 **(D–F)** post-infection. Viral loads were quantified by RT-qPCR in the lungs and normalized by 5000 copies of β-actin **(A–D)**. Neutrophil infiltration in BAL samples was measured by flow cytometry **(B–E)**. Histological slides were stained with H&E and analyzed with 10X magnification **(C–F)**. Data sets are shown as mean +/- SEM. N=4 for each group, one individual experiment. Differences were evaluated by a one-way ANOVA comparing the means of all the columns corresponding to each group, followed by a *post hoc* Tukey test (*p < 0.05; **p < 0.01; ***p < 0.001).

To evaluate the protective capacity of the rBCG-P vaccine, mice were immunized at day 0 and infected with hMPV at day 21 post-immunization. Then the immune response induced by the vaccine was evaluated at days 7 and 28 post-infection (**Supplementary Figure 1A**). Infection was confirmed through the monitoring of disease parameters. Daily weight changes were registered in all the experimental groups (**Supplementary Figure 1B**). The rBCG-P + hMPV group showed significant differences as compared to hMPV group on days 4 and 5. Clinical scores exhibited a similar pattern, suggesting a correlation between both data sets (**Supplementary Figure 1C**). Viral loads were also evaluated for all groups (**Figure 1A**), with significantly lower values detected in rBCG-P+hMPV mice than hMPV mice. However, they were not substantially different from BCG-WT immunized and hMPV-infected mice (BCG-WT + hMPV). In correlation with the viral loads, we found no statistical differences in the total number of infiltrating neutrophils in BALs between the rBCG-P+hMPV mice or the BCG-WT+hMPV mice (**Figure 1B**). However, the neutrophil infiltration in the rBCG-P+hMPV group tended towards decreased values compared to the other groups, similar to those shown by the Mock mice. This decreased infiltration was also related to the histological analyses performed to the lungs of euthanized mice through staining with H&E. We observed that hMPV mice and BCG-WT+hMPV mice exhibited a significant alteration of lung structures and higher cellular infiltration than rBCG-P+hMPV mice, which showed structures similar to those displayed by the Mock mice (**Figure 1C**). Quantification of the histological scores obtained can be found on **Supplementary Figure 1D**. Although it seems that BCG-WT may promote an unspecific immune response after a viral infection with hMPV, we found that our recombinant vaccine potentiates the immune response induced at baseline by the wild-type strain, decreasing the damage after an hMPV-infection. Along these lines, vaccination with the P-protein recombinant BCG could sensitize the immune system of mice at the time of viral infection. This could eventually promote a faster immune response against other components of the virus that are in direct contact with this protein during the viral replication and transcription process using linked recognition as suggested previously (Soto et al., 2018). Overall, these results would indicate that immunization with the rBCG-P vaccine can decrease the disease parameters induced by hMPV at early times of infection.

Since it has been previously described that hMPV exhibits a biphasic infection cycle in mice (Alvarez et al., 2004; Alvarez and Tripp, 2005; Marsico et al., 2018), we evaluated infection parameters at 28 dpi. As expected, viral loads were lower than 7 dpi overall. Remarkably, rBCG-P+hMPV mice showed statistically significant lower levels of viral loads than the hMPV mice (**Figure 1D**). The total number of neutrophils in BAL samples was similar to those found at 7 dpi. Although we observed a tendency towards decreased neutrophil infiltration in the rBCG-P+hMPV group, no statistical differences were found again (**Figure 1E**). Finally, histological slides obtained from rBCG-P+hMPV mice stained with H&E exhibited typical lung structure, associated with decreased cellular infiltration and increased alveolar space (**Figure 1F**). In contrast, hMPV and BCG-WT+hMPV groups showed a more damaged lung structure than the rBCG-P+hMPV and Mock groups (**Figure 1F**). Quantification of the histological scores obtained can be found on **Supplementary Figure 1D**. Thus, our recombinant vaccine prototype protects against hMPV even at later times after the infection.

### Activated Macrophages Induced by the rBCG-P Vaccine Promote a Decrease in the Inflammation at the Lungs in Early and Late Infection Times

**Figure 2:**
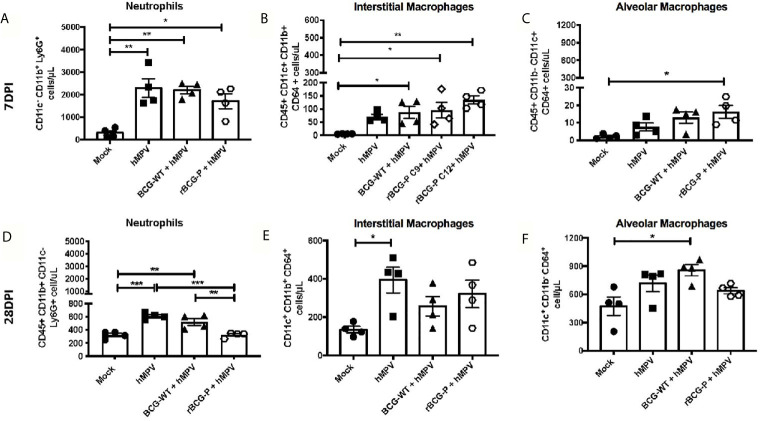
Evaluation of myeloid populations in lungs from hMPV-infected mice at days 7 and 28 post-infection. Several myeloid populations associated with the inflammation and clearance of an hMPV infection were measured at days 7 **(A–C)** and 28 **(D–F)** post-infection by flow cytometry. The populations analyzed were neutrophils **(A, D)**, interstitial macrophages (IM) **(B, E)**, and alveolar macrophages (AM) **(C, F)**. Data sets are shown as mean +/- SEM. N=4 for each group, one individual experiment. Differences were evaluated by a one-way ANOVA comparing the means of all the columns for each group, followed by a *post hoc* Tukey test (*p < 0.05; **p < 0.01; ***p < 0.001).

Although hMPV infection induces a pathology similar to that described for other respiratory viruses, this virus has an enhanced ability to cause severe damage to the lung parenchyma, affecting the gas exchange in the alveoli (Kuiken et al., 2004). We evaluated different myeloid cell populations associated with inflammation and viral clearance (neutrophils and macrophages) at 7 and 28 dpi to assess this damage. The results obtained at 7 dpi show an increase in the number of infiltrating neutrophils in the lungs of all experimental groups infected, as compared to Mock mice (**Figure 2A**). Significantly, a tendency towards lower numbers of neutrophils was observed in the rBCG-P+hMPV group (**Figure 2A**). All immunized and infected mice exhibited higher interstitial macrophages (IM) levels than Mock mice (**Figure 2B**).

Moreover, the rBCG-P+hMPV group promoted a significant increase in the abundance of this subset of macrophages compared to hMPV mice (**Figure 2B**). An increase in the number of alveolar macrophages (AM) in rBCG-P+hMPV mice was also observed compared to Mock mice (**Figure 2C**). The increase detected in the number of IMs could be associated with the decreased neutrophil population saw mainly at 28 dpi.

When these immune populations were evaluated at 28 dpi, a remarkable decrease in the total number of neutrophils in the lung was detected, with similar numbers between the Mock and the rBCG-P+hMPV groups. Here, we found a significant decrease in the neutrophils recruited in the rBCG-P+hMPV group as compared to BCG-WT+hMPV and hMPV groups (**Figure 2D**). Additionally, an increase in the total number of IM and AM was detected for all the groups at 28 dpi as compared to 7 dpi (**Figures 2E, F**). As expected, the significant differences detected at 7 dpi between the rBCG-P+hMPV group and the other groups were not detected at 28 dpi. These results suggest that the rBCG-P vaccine prototype could promote a rapid antiviral immune response, which involves early recruitment of both AM and IM, contributing to hMPV clearance.

### The rBCG-P Vaccine Promotes the Production of Effector Cytokines Upon hMPV Infection

**Figure 3:**
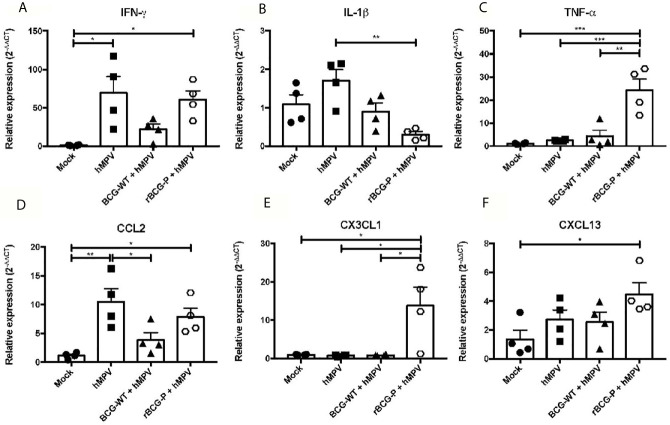
Relative expression (RT-qPCR) of cytokines in lungs from mice immunized with the rBCG-P vaccine upon infection with hMPV at 7 dpi. The relative expression of IFN-γ **(A)**, IL-1β **(B)**, TNF-α **(C)**, CCL2 **(D)**, CXCL13 **(E)**, CX3CL1** (F)** was determined through RT-qPCR from lung RNA samples of mice euthanized at 7 dpi. Data sets are shown as mean +/- SEM. N=4 for each group, one individual experiment. Differences were evaluated by a one-way ANOVA comparing the means of all the columns corresponding to each group, followed by a *post hoc* Tukey test (*p < 0.05; **p < 0.01; ***p < 0.001).

Since the immune response against hMPV is characterized by the secretion of several pro-inflammatory cytokines that fail to promote an immune response capable of efficiently clearing the virus, we evaluated the expression of different cytokines, which could contribute to modulate this phenomenon. The RNA purified from lungs at 7 dpi was used to assess the relative expression of various cytokines and chemokines through RT-qPCR. As IFN-γ is essential for the antiviral immune response, we determined its relative expression. Interestingly, similar expression levels were found between the hMPV group and the rBCG-P+hMPV group (**Figure 3A**), while lower levels were detected in BCG-WT+hMPV and Mock mice. In contrast, we found lower expression levels of IL-1β on the rBCG-P+hMPV group compared to the other groups, but this was statistically different only compared to hMPV mice (**Figure 3B**). TNF-α was strongly expressed in rBCG-P vaccinated animals compared to the other experimental groups studied (**Figure 3C**).

Additionally, three chemokines were analyzed to address the recruitment of monocytes, macrophages, T cells, and B cells into the lungs. CCL2 was measured for promoting the recruitment of monocytes and macrophages, and we found similar expression levels in both hMPV and rBCG-P+hMPV mice (**Figure 3D**) as compared to BCG-WT+hMPV and Mock mice. CX3CL1, a chemokine associated with the recruitment of macrophages and T cells, showed the highest expression levels among the groups (**Figure 3E**). Finally, CXCL13, a chemokine associated with B cell recruitment, exhibited a significant increase in rBCG-P+hMPV mice compared to mock mice and an increase non statistically significant compared to the other groups (**Figure 3F**). Other cytokines associated with different immune polarization profiles (i.e., Th2 and Th17 profiles) were measured. We did not detect a clear response for the induction of the Th2 or Th17 immune response (**Supplementary Figure 2**). Overall, the rBCG-P vaccine decreases the expression levels of cytokines with a suppressive function such as IL-10 and TGF-β1 compared to the hMPV group, but this response was not significant between both groups.

**Figure 4:**
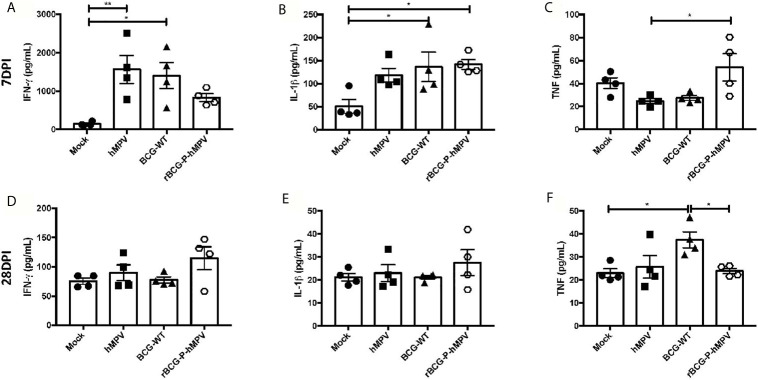
Cytokine response elicited in mice immunized with the rBCG-P vaccine upon infection with hMPV. The secretion of IFN-γ **(A, D)**, IL-1β **(B, E)**, and TNF-α **(C, F)** was performed in the lungs of the mice euthanized at 7 dpi (upper row – **(A–C)**) and 28 dpi (lower row – **(D–F)**). Data sets are shown as mean +/- SEM. N=4 for each group, one individual experiment. Differences were evaluated by a one-way ANOVA comparing the means of all the columns corresponding to each group, followed by a *post hoc* Tukey test (*p < 0.05; **p < 0.01).

Based on the RT-qPCR results obtained, the protein levels of some of these cytokines were evaluated in lung samples at days 7 and 28 post-infection. In addition, no significant differences were detected in the cytokines measured from sera samples except for IFN-γ at 7 dpi and IL-6 at 28 dpi (**Supplementary Figures 3** and **4**), suggesting that hMPV does not induce systemic inflammation, but instead a focalized inflammation in the lungs because very few inflammatory cytokines were found in the blood. Furthermore, these cytokines were not found to be elevated in the sera of animals vaccinated with the rBCG-P strain. We then measured these cytokines in the lungs. The secretion of IFN-γ exhibited a trend towards reduced values in rBCG-P+hMPV mice, compared to BCG-WT+hMPV and hMPV mice at 7 dpi. At 28 dpi, a tendency toward increased levels of IFN-γ was found (**Figures 4A, D**). These changes could be associated with a quick secretion of IFN-γ promoted by the vaccine at early infection times (which then decreases over time), as compared with the other experimental groups. The peak detected at 28 dpi could be associated with the biphasic infection cycle described for hMPV (Alvarez et al., 2004; Alvarez and Tripp, 2005; Marsico et al., 2018). IL-1β was also evaluated due to its direct association with the activation of the immune response by macrophages and its direct link with inflammasome assembly and activation. Subtle differences were found between rBCG-P+hMPV and hMPV mice at 7 dpi (**Figure 4B**), while at 28 dpi, a tendency toward increased levels of IL-1β was found in rBCG-P+hMPV mice, as compared to all the other groups (**Figure 4E**). Because this cytokine is also relevant for controlling the infection caused by hMPV, the link to the biphasic infection cycle could explain these data. TNF was also measured because this cytokine is essential for the control of the viral infection by macrophages. As shown in **Figure 4C**, a quick increase in the secretion of TNF at 7 dpi was observed for that cytokine. However, no such increase was observed at 28 dpi (**Figure 4F**). Additional cytokines, such as IL-6, IL-4, and IL-10, were also evaluated, but no significant differences were found (**Supplementary Figure 5**). Accordingly, low levels of these cytokines have been previously described in BAL samples evaluated at different point times (Guerrero-Plata et al., 2005).

### The rBCG-P Vaccine Induces the Activation of T and B Cells on an Early and Late hMPV Infection

**Figure 5:**
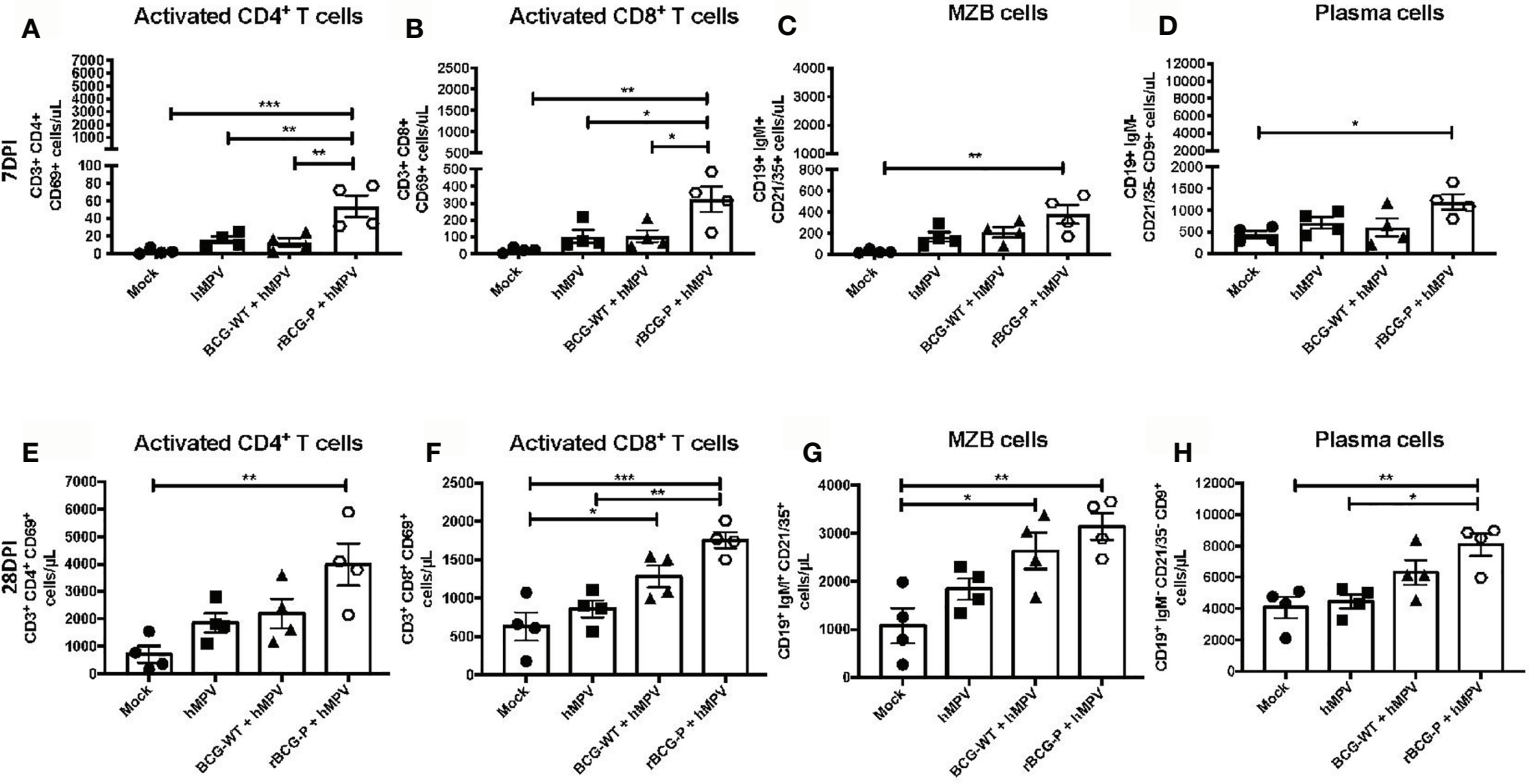
Evaluation of lymphoid populations in the lungs from hMPV-infected animals at 7 and 28 dpi. Different activated T and B lymphocyte populations involved with the clearance of hMPV were measured at days 7 **(A–D)** and 28 **(E–H)** post-infection by flow cytometry. The populations analyzed were CD4^+^ CD69^+^ T cells **(A, E)**, CD8^+^ CD69^+^ T cells **(B, F)**, marginal zone B cells (MZB) **(C, G)**, and plasma cells **(D, H)**. Data sets are shown as mean +/- SEM. N=4 for each group, one individual experiment. Differences were evaluated by a one-way ANOVA comparing the means of all the columns corresponding to each group, followed by a *post hoc* Tukey test (*p < 0.05; **p < 0.01; ***p < 0.001).

It has been previously reported that hMPV promotes an inefficient activation of T cells (González et al., 2017), although it is unknown whether such impairment may also affect B cells. Therefore, we determined if immunization with our recombinant BCG vaccine could induce the proper activation of lymphocytes at different time points (**Figure 5**). Low levels of activated T helper cell populations (CD4^+^ CD69^+^) were found in all the experimental groups at 7 dpi, except for rBCG-P+hMPV mice, which exhibited the highest number of activated T cells (**Figure 5A**). A similar response was observed when the activated cytotoxic T cell populations (CD8^+^ CD69^+^) were measured (**Figure 5B**). We also evaluated the numbers of marginal zone B (MZB) cells due to their contribution to the early activation of T cells and the innate immune response (Attanavanich and Kearney, 2004). We found an expansion of the MZB cell population in the rBCG-P+hMPV group compared to the other groups, but this difference was only significant when compared to the mock group (**Figure 5C**). The differentiated plasma cell population was also evaluated because these cells are the main ones responsible for producing antibodies (Nutt et al., 2015). A similar increase was found for these cells in the rBCG-P+hMPV mice finding significant differences compared to the Mock group at 7 dpi (**Figure 5D**). These results suggest that early activation of B and T cells is induced by administering the rBCG-P vaccine, contributing to the control of the hMPV-induced disease.

Remarkably, when the same immune cells were analyzed at 28 dpi, we found an increase in the total numbers for all the populations measured compared to 7 dpi (**Figures 5E–H**). The most notable rises in cell numbers were always associated with the rBCG-P+hMPV group (**Figures 5E–H**). We detected a marked increase in the levels of activated T helper cells in the rBCG-P+hMPV immunized group, as compared to the other groups, but only a significant difference was found when compared to the Mock group (**Figure 5E**). Surprisingly, the activated cytotoxic T cell population exhibited an expansion in both rBCG-P+hMPV and BCG-WT+hMPV mice compared to Mock mice (**Figure 5F**). However, only the rBCG-P+hMPV group showed statistical differences compared to the hMPV group (**Figure 5F**). Similar results described for the activated cytotoxic T cell populations were detected when the MZB cell expansion was evaluated (**Figure 5G**). However, statistically significant differences were only found between the rBCG-P group and the Mock group. Finally, plasma cell populations significantly increased in the rBCG-P+hMPV group compared to hMPV and Mock mice (**Figure 5H**). These results suggest that our recombinant BCG promotes early and lasting activation of T cells and induces the infiltration of different subsets of B cells to the lungs during an hMPV infection. These immune cells possibly contribute to the amelioration of the hMPV-induced disease, as observed in the rBCG-P-immunized animals. Although we did not find statistically significant differences between the BCG-WT and the rBCG-P, the results suggest that the latter promotes an enhanced activation and expansion of adaptive cells.

### The rBCG-P Vaccine Promotes the Secretion of Mucosal and Serum Immunoglobulins

**Figure 6:**
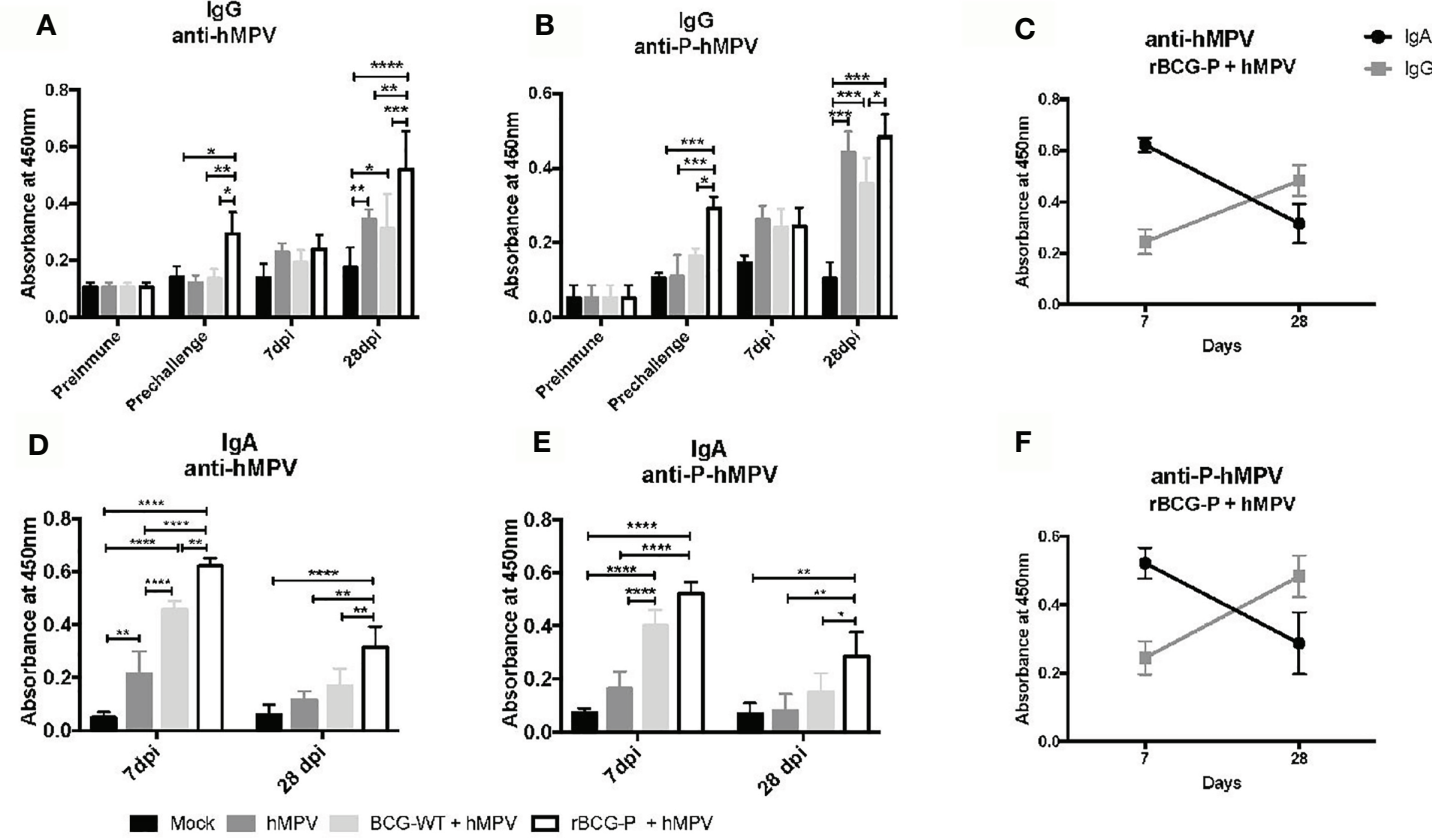
Evaluation of the induction of IgG and IgA specific antibodies against viral antigens upon hMPV infection. IgG level against the virus and the P-hMPV protein were evaluated in serum samples at different times: before immunization (Preimmune), before hMPV infection (Pre challenge), 7- and 28-days post-infection (7 dpi and 28 dpi) (A and B). Specific antibody levels were determined by ELISA assays against hMPV **(A)** or the P-hMPV protein **(B)**. Similarly, IgA levels against the virus and the P-hMPV protein were evaluated in BAL samples at days 7 **(D)** and 28 post-infection **(E)** by ELISA assays. IgA & IgG levels against hMPV **(C)** and the P-hMPV protein **(F)** were graphed for the rBCG-P+hMPV mice. Data sets are shown as mean +/- SEM. N=4 for each group, one individual experiment. Differences were evaluated by a two-way ANOVA followed by a *post hoc* Tukey test (*p < 0.05; **p < 0.01; ***p < 0.001; ****p ≤ 0.0001).

After we characterized the changes of various immune cell populations upon rBCG-P vaccination and hMPV infection, we sought to evaluate whether immunization with a single and low dose of this vaccine could induce the secretion of IgG in serum and IgA in mouse BAL samples (**Figure 6**). The results obtained from the serum samples showed high levels of IgG antibodies against hMPV in the rBCG-P+hMPV group (**Figure 6A**), with levels of antibodies probably associated with the P protein found even before the challenge with the virus. However, these levels were not statistically different at 7 dpi among the different groups. Antibody levels measured for the rBCG-P+hMPV group were higher at 28 dpi than in the other groups (**Figure 6A**). A similar response was observed when the specific antibodies against the P-hMPV protein were evaluated (**Figure 6B**), suggesting an early and a late peak of secretion of IgG antibodies in response to hMPV infection. It is important to note that we found an increase in the antibody levels in the hMPV and the BGC-WT+hMPV mice only at 28 dpi, but not at 7 dpi.

When IgA levels were evaluated in BAL samples, the results differed from those observed in the sera samples. In BALs, high IgA secretion levels against the virus were observed early at 7 dpi in samples from the rBCG-P+hMPV mice compared to the other groups (**Figure 6E**). The BCG-WT+hMPV mice also showed elevated IgA levels compared to the Mock and the hMPV groups. On the other hand, when the IgA levels were evaluated at 28 dpi, the rBCG-P+hMPV group showed a statistically significant increase in their levels compared with all the other groups (**Figure 6D**). Similar responses as the one seen in the anti-hMPV antibodies were found when the anti-P-hMPV antibodies were measured, detecting an increase in the IgA levels at 7 dpi only in the BCG-WT+hMPV and the rBCG-P+hMPV groups (**Figure 6E**). The BCG-WT group may have detectable antibodies against the P protein due to the measurement time was after the hMPV infection. These antibody levels were evaluated after viral infection. Despite this, we found that the rBCG-P induced significantly higher levels of P protein-specific antibodies, as well as total antibodies against the virus. Increased secretion of antibodies specific for antigens other than the P protein may be mediated by a mechanism known as linked recognition, as previously described (Soto et al., 2018).

We compared the secretion kinetics of IgG and IgA induced by the rBCG-P+hMPV group to determine whether there could be a significant pattern in these secretions. We identified an increase in the total anti-hMPV IgA levels in the rBCG-P+hMPV group at early times (day 7) compared to the IgG levels (**Figure 6C**). However, at 28 dpi, we observed that IgA levels decreased while IgG levels increased (**Figure 6C**), suggesting that IgA could play a relevant role early in the infection, while the IgG effect could be mainly associated with the late course of the disease or the viral clearance. Similar responses were found when we evaluated the secretion kinetics of IgG and IgA induced by the rBCG-P+hMPV group for the antibodies against the P-hMPV protein (**Figure 6F**). A similar effect was only observed in the BCG-WT+hMPV group (**Supplementary Figure 6**).

These results suggest that our vaccine candidate can induce the secretion of anti-hMPV and anti-P antibodies at early (7 dpi) and late (28 dpi) timepoints of infection with hMPV, yielding an early secretion of IgA and the late secretion of IgG. Remarkably, we only detected specific antibodies prior to the viral infection in rBCG-P immunized mice, suggesting that the observed response is a product of the rBCG-P vaccine and not due to unspecific activation of the immune system, which the BCG-WT may induce (Miyaji et al., 2001; Hosseini et al., 2015; Soto et al., 2018).

### Transfer of T Cells or Sera From rBCG-P Immunized Mice Protects Against hMPV Infection

**Figure 7:**
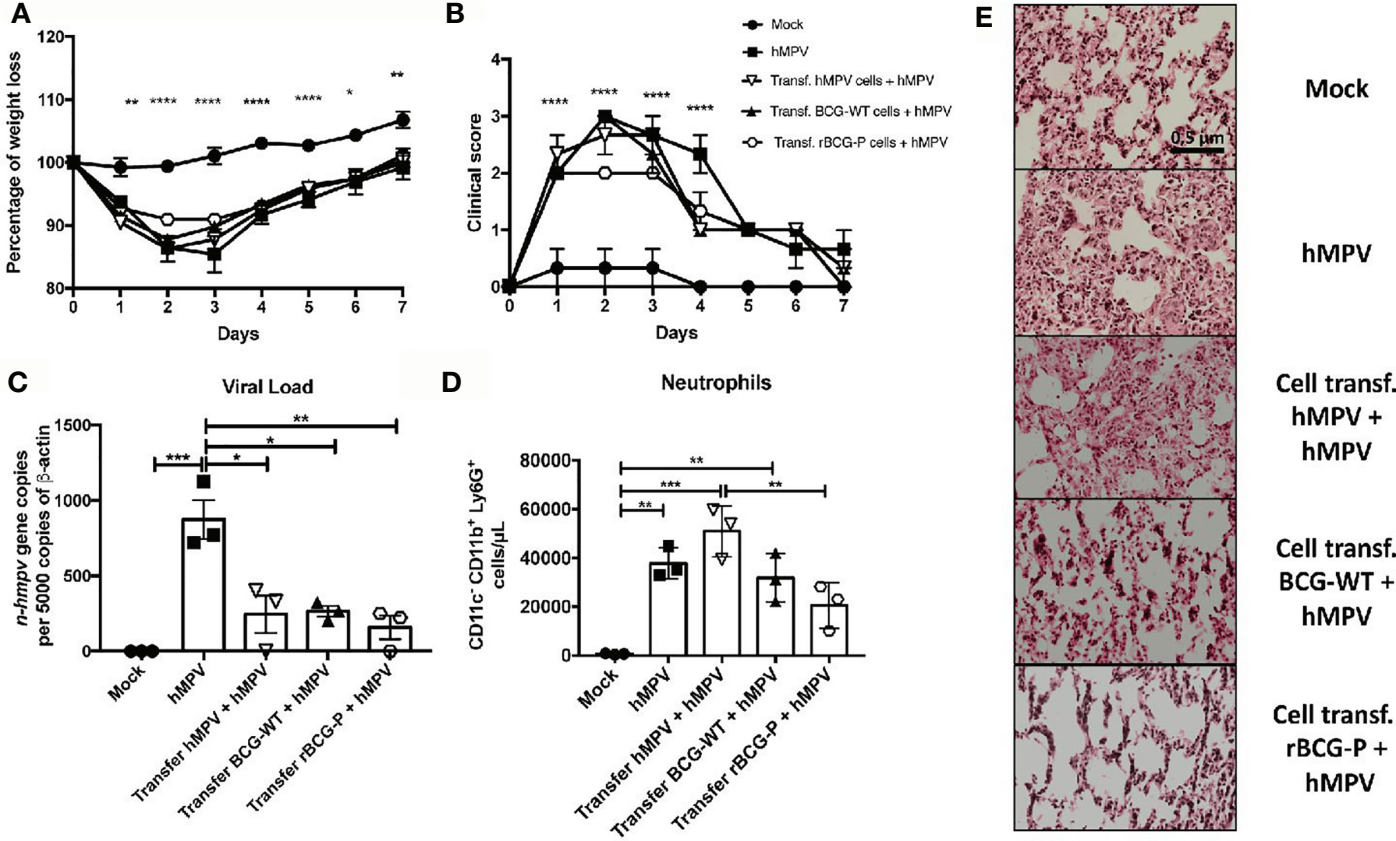
Cell transfer from mice previously immunized the rBCG-P decreases the hMPV-associated pathology in naïve mice. T cells from 7 days post-infection were passively transferred to naïve mice. Then, mice were infected with hMPV. As controls, a non-transferred group was also infected (hMPV group), and a non-infected (Mock) group was included. Disease parameters, such as weight loss **(A)** and clinical score **(B)**, were evaluated. Also, the viral load in the lungs **(C)**, neutrophil infiltration to the BAL **(D)**, and histopathological lung H&E staining **(E)** were evaluated. Data sets are shown as mean +/- SEM. N=3 for each group, one individual experiment. Differences were evaluated by a one-way ANOVA comparing the means of all the columns for each group, followed by a *post hoc* Tukey test (*p < 0.05; **p < 0.01; ***p < 0.001; ****p ≤ 0.0001).

To determine the contribution of antibodies and T cells induced by immunization with the rBCG-P vaccine in protecting against hMPV, total T cells or serum were obtained from vaccinated and infected mice and then adoptively transferred to naïve mice, as described in the material and methods section and **Supplementary Figure 7**. For the T cell transfer assay, only the mock-treated (Mock) group did not exhibit any weight loss compared with all the other hMPV-infected experimental groups (**Figure 7A**). We observed that all the hMPV-infected groups suffered a weight loss and a posterior weight recovery through time, but animals transferred from the rBCG-P + hMPV group showed the least weight loss within the infected groups. The clinical scores were similar in all hMPV infected groups except in the group transferred with rBCG-P + hMPV cells, which reached levels similar to those of the Mock group (**Figure 7B**). Viral loads were measured in the lungs of the transferred mice. We found that the non-transferred but hMPV-infected (hMPV) group exhibited the highest viral genetic material than all the other experimental groups (**Figure 7C**). The number of neutrophils recruited into the lungs was mostly similar in all hMPV-infected mice (**Figure 7D**). Mice transferred with cells from hMPV infected mice and then infected with hMPV (Transf. hMPV cells + hMPV) had the highest number of infiltrating cells (**Figure 7D**). Mice transferred with cells from the rBCG-P+hMPV group and then infected with hMPV (Transf. rBCG-P cells + hMPV) showed a decrease in the numbers of neutrophils as compared to the Transf. hMPV cells + hMPV mice (**Figure 7D**). Slides stained with H&E displayed high infiltration of cells and significant lung structure loss in the hMPV, the Transf. hMPV cells + hMPV, and the Transf. BCG-WT cells + hMPV groups. In contrast, the Transf. rBCG-P cells + hMPV mice showed a minor loss of lung structure and cellular infiltration, with characteristics similar to those seen in the Mock group (**Figure 7E**), suggesting that cells from rBCG-P immunized mice may play a protective role during an hMPV infection. Quantification of the histological scores obtained can be found on **Supplementary Figure 7B**.

**Figure 8:**
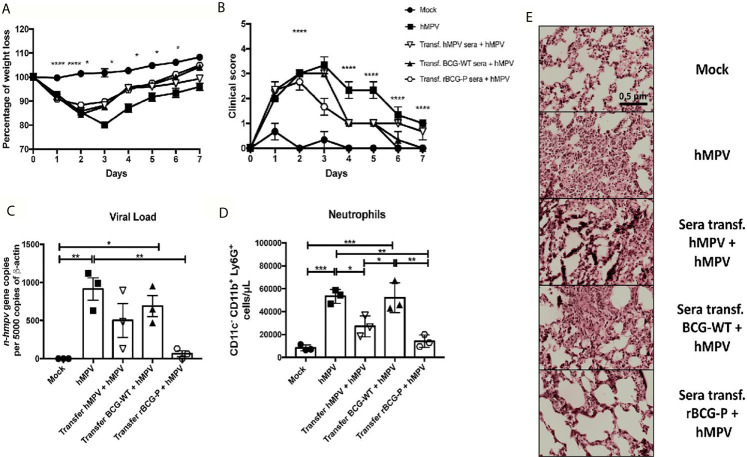
Sera transfer from mice previously immunized with the rBCG-P protects naïve mice from hMPV-associated pathology. Sera from mice of the previous experimental groups at 7 days post-infection were passively transferred to naïve mice. Then, transferred mice were infected with hMPV. As controls, a non-transferred group was also infected (hMPV group), and a non-infected (Mock) group was included. Disease parameters, such as weight loss **(A)** and clinical score **(B)**, were evaluated. Also, the viral load in the lungs **(C)**, neutrophil infiltration to the BAL **(D)**, and histopathological lung H&E staining **(E)** were evaluated. Data sets are shown as mean +/- SEM. N=3 for each group, one individual experiment. Differences were evaluated by a one-way ANOVA comparing the means of all the columns for each group, followed by a *post hoc* Tukey test (*p < 0.05; **p < 0.01; ***p < 0.001; ****p ≤ 0.0001).

When sera transfer assays were performed, we found that the animals transferred with serum from the rBCG-P+hMPV and the BCG-WT+hMPV group recovered weight faster than the other groups, while the Mock group did not lose any weight (**Figure 8A**). Also, we determined that the animals transferred with sera from the hMPV infected mice showed a slower weight recovery in the time, as compared to the other groups (**Figure 8A**). Clinical scores showed that the Mock group and the mice transferred with the serum from the rBCG-P+hMPV group were the only ones with no signs of illness at 7 dpi (**Figure 8B**). Quantification of viral loads showed a significant decrease in the mice transferred with sera from the rBCG-P+hMPV group compared with the non-transferred but hMPV infected group (**Figure 8C**). To correlate the viral loads with the lung inflammation, neutrophil populations infiltrated into the lungs were measured (**Figure 8D**). We found a decrease in the number of this immune population in the mice transferred with sera from the rBCG-P+hMPV group, significantly lower than those detected for the hMPV group and the mice transferred with sera from the BCG-WT+hMPV group (**Figure 8D**). Surprisingly, mice transferred with sera from the hMPV + hMPV-infected group decreased their neutrophils values in the BAL (**Figure 8D**). Finally, staining of cuts with H&E showed similar lung structures and low infiltration between the Mock group and the mice transferred with sera from the rBCG-P+hMPV group. In contrast, high levels of lung structure disruption were observed in the mice transferred with sera from the BCG-WT+hMPV infected mice and the only hMPV-infected mice (**Figure 8E**). Quantification of the histological scores obtained can be found on **Supplementary Figure 7C**.

Altogether, these results suggest that vaccination with the rBCG-P vaccine prototype induces the proliferation of T cells and the secretion of antibodies that can control the infection caused by hMPV, promoting a protective and safe response against hMPV, as compared with the BCG-WT strain. This response was mainly characterized by less weight loss, infiltration of inflammatory cells in the tissue, and lower viral loads. Remarkably, despite BCG-WT shows a positive effect promoting an immune response able to partially protect against hMPV infection, data obtained in this study support the notion that rBCG-P induces with better efficiency an innate and adaptive immune response capable of protecting against hMPV infection, upon administration of only one dose of this vaccine.

## Discussion

The human metapneumovirus (hMPV) is an emergent virus associated with acute lower respiratory tract infections (ALRTI). hMPV is considered the second most relevant respiratory virus responsible for promoting bronchiolitis and pneumonia (Kahn, 2006; Schildgen et al., 2011) and has been associated with an exacerbated asthma response (Williams et al., 2005). It is currently considered that the infection induced by hMPV in children under one year old is more lethal than the one reported for other viruses, such as hRSV (Williams, 2005). Nowadays, one of the main concerns regarding hMPV is the lack of knowledge underlying the immune evasion mechanisms used by this virus (Céspedes et al., 2013) and its capacity to promote a secondary or biphasic infection, leading to the detection of viral genetic material even up to 60 days after the initial infection (Alvarez et al., 2004; Alvarez and Tripp, 2005). The infection with hMPV promotes a disease associated with an increase in the secretion of pro-inflammatory cytokines, the polarization of CD4^+^ T cells into a Th2-like profile, an increase in the infiltration of neutrophils into the lung, and an inefficient T and B cell function, which impairs viral clearance (Peiris et al., 2003; Ito et al., 2004; Romagnani, 2006; Hamelin et al., 2006; Fietta and Delsante, 2009). This inadequate B cell response may contribute to the limited B cell repertoire found in newborns (up until 6 months of age), which is related with low somatic hypermutation of the immunoglobulin genes (Williams et al., 2009).

In this work, we evaluated the protective immunity against hMPV infection elicited by a single and low dose of a rBCG-P vaccine derived from the master cell bank stage of the cGMP process. The BCG-WT strain used in this study was grown with OADC supplementation, which is usually an essential nutritional requirement for these mycobacteria. This variable is not likely associated with the enhanced protective response reported by the rBCG-P strain, which is grown without OADC during the master cell bank cGMP production process. The absence of OADC in the recombinant vaccine suggests that all the effects reported in this study are directly related to the immunogenicity induced by the vaccine. Besides, we evaluate the safety of the vaccine, confirmed that the rBCG is not present on other tissues besides the immunization site. The dissemination was evaluated in the spleens from mice (data not shown). These results are consistent with observations made in a human clinical study for another recombinant BCG vaccine strain generated under the same conditions and evaluated (Abarca et al., 2020). As part of this study, no BCG was found in saliva, urine and blood samples from the enrolled subjects (Abarca et al., 2020).

Since a natural hMPV infection induces inflammation in the lungs, prompting an increase in neutrophil recruitment and the secretion of various cytokines and chemokines, there is evident damage to the respiratory epithelial architecture, with the loss of ciliation and increased mucus secretion as the acute symptoms of disease (Vargas et al., 2004; Hamelin et al., 2006). This type of inflammation has also been associated with the presence of viral proteins in the pulmonary parenchyma, triggering a robust immune response against hMPV by alveolar epithelial cells (AECs), DCs, and AMs.

In this work, we observed that AMs resident in the lung could be involved in the resolution and the modulation of the inflammation due to their capacity of inducing type I IFN (α/β), which participates in the antiviral response (Kolli et al., 2014), and other molecules as TNF-α. However, previous reports suggested that AMs increased in response to hMPV infection, inducing the exacerbation of the disease (Kolli et al., 2014). Depletion of AMs resulted in a reduced clinical score upon infection with hMPV compared with non-depleted mice, therefore suggesting that the disease was less severe in the absence of AMs (Kolli et al., 2014). We detected an opposite response in our work, which could be partially explained by high amounts of IMs. It has been reported that IMs contribute to promoting an anti-inflammatory environment, associated with a Th17-like immune response, led mainly by the secretion of IL-10 (Kawano et al., 2016; Schyns et al., 2018). Still, our data did not show differences in the evaluated time points either for the relative expression of the genes nor for the secreted proteins (**Supplementary Figures 2** and **5**). This profile might regulate a possible imbalance of inflammatory responses, therefore controlling the exacerbated lung damage. Since IMs are associated with lung remodeling regulation by inhibiting metalloproteinases (Ferrari-Lacraz et al., 2001), an increase of this immune population may have an active role in the associated immunopathology.

Another role associated with the AMs is the expression of PD-L1 during hMPV infections, which modulates T cell activation (Hastings et al., 2015). Some studies have shown that the expression of PD-L1 induces the inhibition of the activated CD8^+^ T cell population during hMPV infection, affecting the antiviral immune response (Erickson et al., 2012; Hastings et al., 2015). Therefore, we suggest that the presence of resident AMs in the lung of these animals affect this adaptive immune response, resulting in a decreased level of activation of CD4^+^ and CD8^+^ T cell populations in mice naturally infected with hMPV. Interestingly, this effect was not found in the rBCG-P+hMPV group, which showed higher levels of AMs when compared to the other experimental groups and showed high amounts of activated CD4^+^ and CD8^+^ T cell populations (**Figure 5**). These results may suggest that the rBCG-P vaccine could be modulating the expression of PD-L1 in the context of an hMPV infection, favoring the development of an efficient and adequate adaptive immune response. However, this remains to be evaluated. Additionally, high levels of relative expression of the chemokine CX3CL1 were found (**Figure 3**), enhancing the recruitment of T cells into the lungs (**Figure 5**).

Previous reports suggest that CD8^+^ T cells can promote viral clearance even in the absence of helper T cells and B cells (Kolli et al., 2008). Interestingly, many activated T and B cells were detected in the vaccinated mice (**Figure 5**). An increased expression of the chemokines CX3CL1 and CXCL13, which promote the recruitment of T cells and B cells, was also detected (**Figure 5**). In this work, we found that total T cells transferred to naïve mice showed a positive effect on the viral clearance, with subtle differences in the decrease of viral loads and recruited neutrophils and less damage to the lung compared to the other infected groups (**Figure 7**). However, the sera transferred to naïve mice showed enhanced protective capacities (**Figure 8**), suggesting that B cells are relevant for resolving the viral infection.

The role of B cells has been directly associated with pulmonary damage induced by a primary hMPV infection, suggesting that these cells fail to control the infection, causing a sub-optimal antibody production. However, it has been reported that when B cells are transferred to naïve mice, they promote partial protection against hMPV infection (Alvarez et al., 2004; Alvarez and Tripp, 2005). Here, immunization with the rBCG-P vaccine induced the differentiation of B cells, promoting a strong response of MZB cells, whose effect is mainly associated with a quick response against infections, stimulating the secretion of IgM and IgG antibodies as the first line of defense (Won and Kearney, 2002; Zouali and Richard, 2011). Furthermore, MZB cells are a type of B cells considered innate-like B cells that might promote the priming of CD4^+^ T cells (Kearney, 2008). MZB cells have been described to be found mainly in the spleen and rarely in the lymph nodes (Koike et al., 1996). Surprisingly, we found MZB cells in lungs early at 7 dpi, but only for the rBCG-P+hMPV group. Interestingly, the number of MZB cells increased at 28 dpi in all the BCG immunized mice (**Figure 5**).

Moreover, we found high amounts of plasma cells mainly in the rBCG-P+hMPV group (**Figure 5**), which correlated with high anti-hMPV and anti-P antibody levels observed early and late after the infection, compared to the mice infected but not immunized (**Figure 6**). These results suggest that the rBCG-P vaccine can change the typical antibody profile associated with the hMPV disease, triggering an adequate viral clearance accompanied by T and B cell activation.

As mentioned above, hMPV can promote a deficient antibody secretion associated with a low protective role (Alvarez et al., 2004). However, it was reported that different strategies for developing vaccines might need to stimulate an increase in antibody levels. A recent publication demonstrates that a formulation including the hMPV nucleoprotein (N-hMPV) with AbISCO-100 adjuvant can increase IgG, IgG1, and IgG2a isotype antibodies (Diaz-Dinamarca et al., 2018). Other strategies to improve the antibody secretion were evaluated using a live-attenuated virus, where they detected an antibody response 4 weeks post-infection (Buchholz et al., 2006). Here, we found IgA and IgG antibodies against the full virus and the P-hMPV protein. Unexpectedly, we found an increase in the IgA levels early at day 7 post-infection, mainly in the rBCG-P+hMPV group and a minor IgG level. However, when the IgA levels were evaluated on day 28, we found a decrease in this immunoglobulin and increased IgG antibodies (**Figure 6**).

Finally, the transfer of both cells and sera showed that immunization with the rBCG-P vaccine could modulate the viral clearance in the lung. However, these data suggest that the antibodies induced from rBCG-P+hMPV immunized mice can promote a better viral clearance than cells obtained from the same group. Interestingly, the sera transferred from hMPV-infected mice into naïve mice was enough to promote a decrease in the number of neutrophils recruited to the lungs and decrease the tissue damage slightly, similarly to what has been reported previously (Soto et al., 2018).

## Conclusions

hMPV is a significant respiratory pathogen affecting vulnerable populations, with a high economic and social burden worldwide. Therapies against this virus must be generated soon to address the impact of this virus. Here, we showed that a single and low dose of a recombinant BCG strain expressing the phosphoprotein of hMPV promoted a protective and well-balanced immune response. This response induced the activation and infiltration of different populations of innate cells and the activation of helper and cytotoxic T lymphocytes. We also identified high recruitments of B cells and the secretion of antibodies in the lungs, which could contribute to viral clearance, by promoting the secretion of mucosal and serum immunoglobulins that might be able to protect against an hMPV infection. Therefore, the rBCG-P vaccine prototype is a promising candidate to modulate the unbalanced immune response elicited upon hMPV infection. Because this protective effect was achieved with a single and low dose, advancement towards clinical trials is also a promising prospect.

## Data Availability Statement

The original contributions presented in the study are included in the article/**Supplementary Material**. Further inquiries can be directed to the corresponding author.

## Ethics Statement

All mouse experiments were conducted in agreement with ethical standards and according to the local animal protection law number 20.800. All experimental protocols were designed according to the Sanitary Code of Terrestrial Animals of the World Organization for Animal Health (OIE, 24^a^ Edition, 2015) and were reviewed and approved by the Scientific Ethical Committee for Animal and Environment Care of the Pontificia Universidad Católica de Chile (Protocol number 160819006 for rBCG-P-hMPV).

## Author Contributions

JS: conceptualization, experimental development, writing original draft, figure design, review, editing, and revision of all versions. NG: conceptualization, experimental development, review, editing, and revision of all versions. GP: experimental development, editing, and revision of all versions. GC-M: experimental development and revision of all versions. SB: conceptualization, revision of original draft, editing, and revision of final version. AK: conceptualization, revision of initial draft, editing, and revision of final version. All authors contributed to the article and approved the submitted version.

## Funding

Millennium Institute of Immunology and Immunotherapy (P09/016-F, ICN09_016, AMK and SMB); CORFO grant #13CTI-21526/P4 and P5; CONICYT/FONDECYT grants #3190590 (JAS); #1190830 (AMK); #1170964 SMB), CONICYT scholarship #21190183 (NMSG). This work was also supported by the Regional Government of Antofagasta through the Innovation Fund for Competitiveness FICR 2017 (BIP Code: 30488811-0). AMK is a Helen C. Levitt Visiting Professor at the Department of Microbiology and Immunology of the University of Iowa.

## Conflict of Interest

A patent has been issued to SB and AK for the rBCG-P vaccine in several countries [Number 201302829, Chile].

The authors declare that the research was conducted in the absence of any commercial or financial relationships that could be construed as a potential conflict of interest.

## Supplementary Material

Supplementary Figure 1 Experimental set up for the immunization of the mice and disease parameters. The immunization scheme indicating the times of immunization and harvest of samples **(A)**, the changes of body weight **(B)** and clinical scores **(C)** are depicted. The histological clinical quantification **(D)** was determinate using three histological pictures from 3 mouse. Data sets are shown as mean +/- SEM. N=4 for each group, one individual experiment **(B, C)**. Differences were evaluated by a multi-test followed by a *post hoc* Tukey test (*=p<0.05; **=p<0.01; ***=p<0.001; ****=p ≤ 0.0001). Data sets are shown as mean +/- SEM. N=4 for each group, one individual experiment **(D)**. Differences were evaluated by a two-way ANOVA followed by a *post hoc* Tukey test (*=p<0.05; **=p<0.01; ***=p<0.001; ****=p ≤ 0.0001).

Supplementary Figure 2 Relative expression of cytokines in lungs from mice immunized with the rBCG-P vaccine upon infection with hMPV at 7 dpi. The relative expression of IL-4 **(A)**, IL-6 **(B)**, IL-10 **(C)**, TGF-β1**(D)**, IL-12p40 **(E)**, IL-17 **(F)** was performed from lung RNA of the mice euthanized at 7 dpi. Data sets are shown as mean +/- SEM. N=4 for each group, one individual experiment. Differences were evaluated by a one-way ANOVA comparing the means of all the columns for each group, followed by a *post hoc* Tukey test (*=p<0.05; **=p<0.01).

Supplementary Figure 3 Cytokine secreted in sera from mice immunized with the rBCG-P vaccine upon infection with hMPV at 7 dpi. The secretion of IFN-γ **(A)**, IL-1β **(B)**, TNF **(C)**, IL-4 **(D)**, IL-6 **(E)**, IL-10 **(F)** was performed in the serum of the mice euthanized at 7 dpi. Data sets are shown as mean +/- SEM. N=4 for each group, one individual experiment. Differences were evaluated by a one-way ANOVA comparing the means of all the columns for each group, followed by a *post hoc* Tukey test (*=p<0.05; **=p<0.01).

Supplementary Figure 4 Cytokine secreted in sera from mice immunized with the rBCG-P vaccine upon infection with hMPV at 28 dpi. The secretion of IFN-γ **(A)**, IL-1β **(B)**, TNF **(C)**, IL-4 **(D)**, IL-6 **(E)**, IL-10 **(F)** was performed in the serum of the mice euthanized at 28 dpi. Data sets are shown as mean +/- SEM. N=4 for each group, one individual experiment. Differences were evaluated by a one-way ANOVA comparing the means of all the columns for each group, followed by a *post hoc* Tukey test (*=p<0.05; **=p<0.01).

Supplementary Figure 5 Cytokine secreted in mice immunized with the rBCG-P vaccine upon infection with hMPV. Evaluation of the secretion of IL-4 **(A, D)**, IL-6 **(B, E)**, and IL-10 **(C, F)** was performed in the lungs of the mice euthanized at 7 dpi (upper row – **(A–C)**_ and 28 dpi (lower row – **(D–F)**). Data sets are shown as mean +/- SEM. N=4 for each group, one individual experiment. Differences were evaluated by a one-way ANOVA comparing the means of all the columns for each group, followed by a *post hoc* Tukey test (*=p<0.05; **=p<0.01).

Supplementary Figure 6 Correlation of IgG and IgA specific antibodies against viral antigens after an hMPV infection. The correlation of the IgA/IgG ratio against hMPV **(A–C)** and the P-hMPV protein **(D–F)** was graphed for the mock-treated **(A, D)**, hMPV infected **(B, E)**, and BCG-WT+hMPV **(C, F)** groups. Data sets are shown as mean +/- SEM. N=4 for each group, one individual experiment.

Supplementary Figure 7 Experimental set up for the immunization of the mice for the transfer assays and histological clinical quantification. The experimental set up shows the times of immunization and harvest of samples for the initial experimental set up (upper segment) and the later T cell and sera transfer (lower segment) **(A)**. The histological clinical quantification of the cell transfer assay **(B)** and sera transfer assay **(C)** were determinate using three histological pictures from 3 mouse. Differences were evaluated by a one-way ANOVA comparing the means of all the columns for each group, followed by a *post hoc* Tukey test (*=p<0.05; **=p<0.01).
