# Supplementary figures and images for: Induction of Protective Immunity by a Single Low Dose of a Master Cell Bank cGMP-rBCG-P Vaccine Against the Human Metapneumovirus in Mice
Source: Front Cell Infect Microbiol. 2021 Jun 29;11:662714. doi: 10.3389/fcimb.2021.662714 (PMC8276701; doi:10.3389/fcimb.2021.662714)

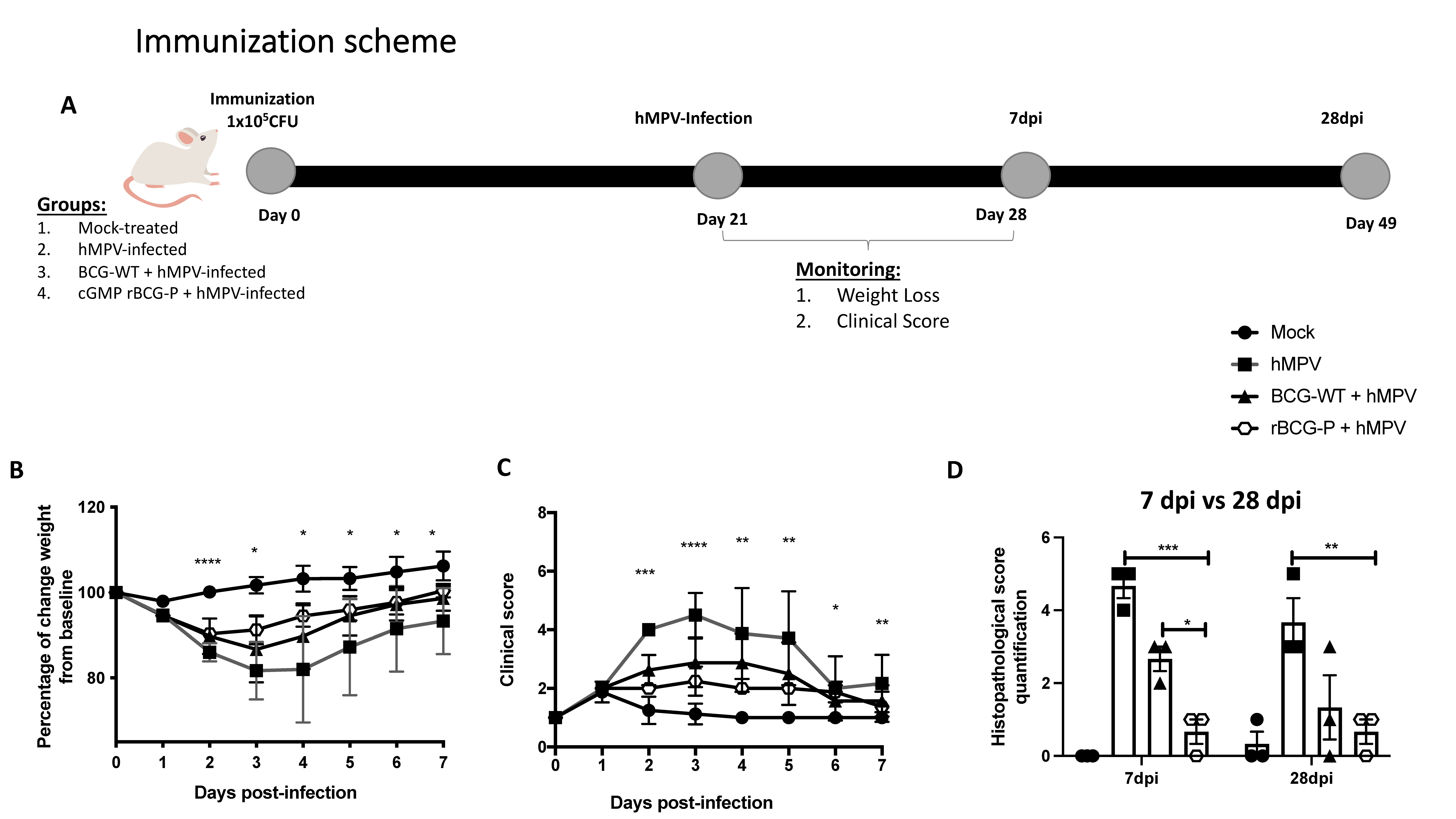

Supplement: Supplementary Figure 1 — Experimental set up for the immunization of the mice and disease parameters. The immunization scheme indicating the times of immunization and harvest of samples (A), the changes of body weight (B) and clinical scores (C) are depicted. The histological clinical quantification (D) was determinate using three histological pictures from 3 mouse. Data sets are shown as mean +/- SEM. N=4 for each group, one individual experiment (B, C). Differences were evaluated by a multi-test followed by a post hoc Tukey test (*=p<0.05; **=p<0.01; ***=p<0.001; ****=p ≤ 0.0001). Data sets are shown as mean +/- SEM. N=4 for each group, one individual experiment (D). Differences were evaluated by a two-way ANOVA followed by a post hoc Tukey test (*=p<0.05; **=p<0.01; ***=p<0.001; ****=p ≤ 0.0001). [file Image_1.tiff]

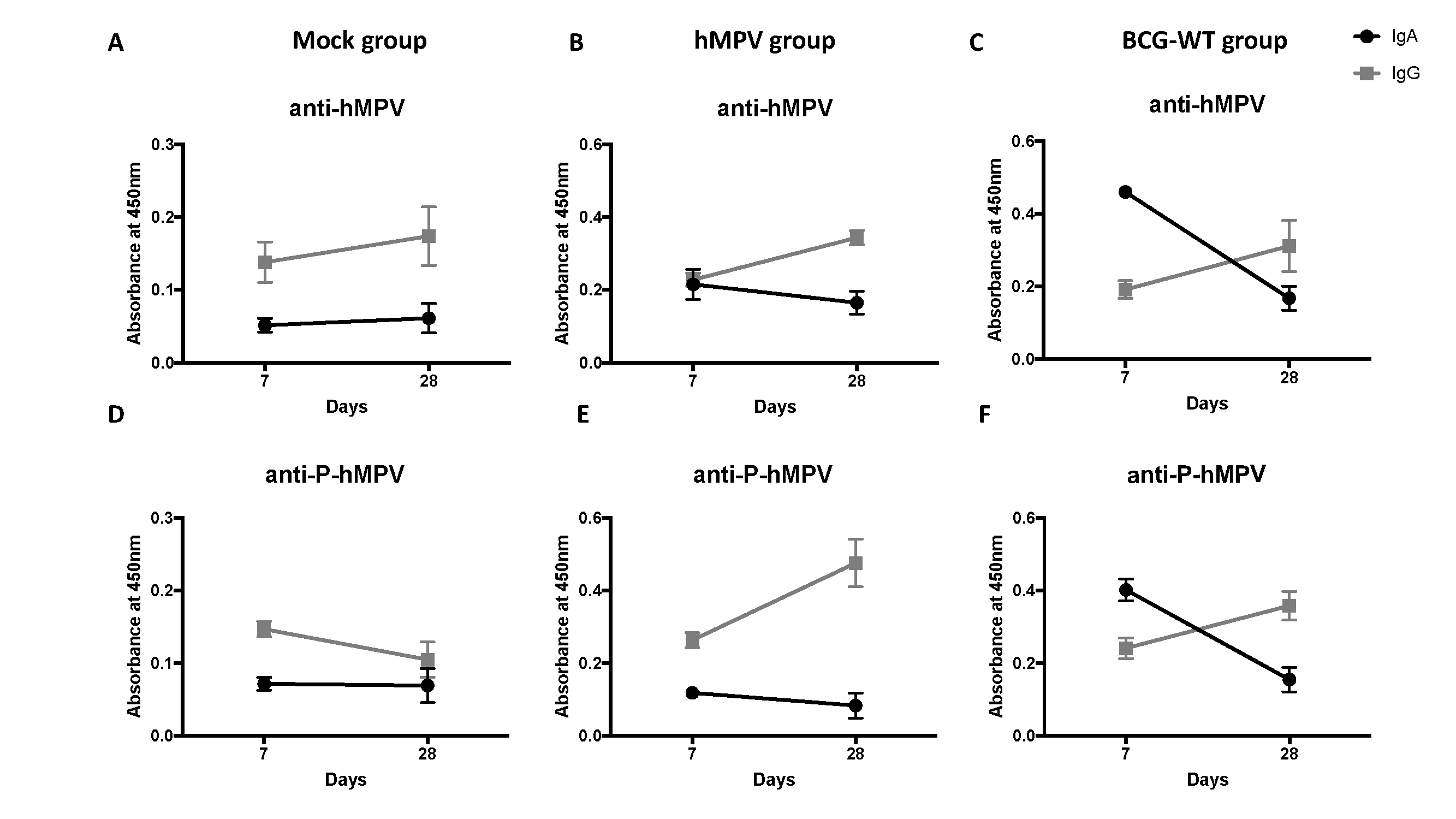

Supplement: Supplementary Figure 6 — Correlation of IgG and IgA specific antibodies against viral antigens after an hMPV infection. The correlation of the IgA/IgG ratio against hMPV (A–C) and the P-hMPV protein (D–F) was graphed for the mock-treated (A, D), hMPV infected (B, E), and BCG-WT+hMPV (C, F) groups. Data sets are shown as mean +/- SEM. N=4 for each group, one individual experiment. [file Image_6.tiff]
